# GANs for Medical Image Synthesis: An Empirical Study

**DOI:** 10.3390/jimaging9030069

**Published:** 2023-03-16

**Authors:** Youssef Skandarani, Pierre-Marc Jodoin, Alain Lalande

**Affiliations:** 1 ImViA Laboratory, University of Bourgogne Franche-Comte, 21000 Dijon, France; 2 CASIS Inc., 21800 Quetigny, France; 3 Department of Computer Science, University of Sherbrooke, Sherbrooke, QC J1K 2R1, Canada; 4 Department of Medical Imaging, University Hospital of Dijon, 21079 Dijon, France

**Keywords:** GAN, MRI, CT, heart, retina, liver, adversarial

## Abstract

Generative adversarial networks (GANs) have become increasingly powerful, generating mind-blowing photorealistic images that mimic the content of datasets they have been trained to replicate. One recurrent theme in medical imaging, is whether GANs can also be as effective at generating workable medical data, as they are for generating realistic RGB images. In this paper, we perform a multi-GAN and multi-application study, to gauge the benefits of GANs in medical imaging. We tested various GAN architectures, from basic DCGAN to more sophisticated style-based GANs, on three medical imaging modalities and organs, namely: cardiac cine-MRI, liver CT, and RGB retina images. GANs were trained on well-known and widely utilized datasets, from which their FID scores were computed, to measure the visual acuity of their generated images. We further tested their usefulness by measuring the segmentation accuracy of a U-Net trained on these generated images and the original data. The results reveal that GANs are far from being equal, as some are ill-suited for medical imaging applications, while others performed much better. The top-performing GANs are capable of generating realistic-looking medical images by FID standards, that can fool trained experts in a visual Turing test and comply to some metrics. However, segmentation results suggest that no GAN is capable of reproducing the full richness of medical datasets.

## 1. Introduction

During the last decade, machine learning has been widely adopted, mainly due to the advent of deep neural networks and their state-of-the-art results on a variety of medical imaging tasks. Meanwhile, the introduction of generative adversarial networks (GANs) by [1], drove generative modeling and data synthesis to levels of quality never before achieved. The research on GANs grew at an ever increasing pace, with each iteration pushing back the limits of image quality. Perhaps, one notable breakthrough in image quality came from [2] and their *Big GAN*. Not so long after, another drastic jump in the quality and diversity of generated images came with *Style GAN* [3], which exhibited highly realistic high-resolution human faces. Motivated by the impressive results achieved by GANs on natural images, the goal of this work is to evaluate how well these machines perform on medical data, an area well-known for its smaller datasets and strict anatomical requirements. Recent reviews have been published, analyzing the use of GANs in medical image analysis [4,5,6]. The distinctiveness of our work is the empirical evaluation of the benefits of GAN-generated data in this context, in addition to the large hyperparameters analysis of the different approaches.

### 1.1. Medical Image Analysis

Medical image analysis aims to un-invasively extract information about a patient’s medical condition. Medical images are images acquired from one of multiple modalities, be it magnetic resonance imaging (MRI), computed tomography (CT), positron emission tomography (PET), or ultrasound (US), to name a few. The acquired images are generally processed using image analysis and/or computer vision techniques, to extract certain useful information about the data at hand, for example, to classify whether the case is normal or pathological. One of the most routine tasks in clinical practice is image contouring, or segmentation. Image segmentation is the operation of outlining parts of the images that belong to certain classes of interest. For example, in the case of cardiac MRI, one may delineate the left ventricular cavity and myocardium, with the objective of measuring blood volumes and contraction rates.

In recent years, machine learning and deep learning garnered a large interest from the medical imaging community, due to their unprecedented achievements in a large swath of computer vision tasks. However, machine learning software have not yet been widely adopted in clinical practice, largely due to the fact that neural networks are still error prone under certain conditions (domain adaptation, different acquisition protocols, missing data, etc). One reason for this, derives from the fact that fully-annotated medical imaging datasets are much smaller than those in other areas. For example, the gold standard computer vision ImageNet [7] dataset, contains more than 14 million annotated images, while a typical medical image dataset is three to four orders of magnitude smaller. This is because the creation of medical imaging datasets is costly and difficult, due to the sensitive nature of the data and the highly specific domain knowledge required to reliably annotate it. The paucity of training data in medical imaging, has made the search for other means of acquiring training sets an active area of research [8].

### 1.2. Synthetic Data and Medical Imaging

Recently, GANs have received growing attention from the medical research community, with the hope of using them to synthesize realistic-looking medical images. For example, [9] trained a GAN to synthesis new T1-weighted brain MRIs, with comparable quality to real images, and [10] succeeded in generating high resolution skin lesion images which experts could not reliably tell apart from real images. In [11], they took advantage of GANs to generate brain MRIs that achieved high scores both in qualitative and quantitative evaluation. In [12], the authors showed that GAN-generated images of lung cancer nodules are nearly indistinguishable from real images, even by trained radiologists.

GANs were also used as a means for generating more training data. In [13], the authors trained a GAN to generate synthetic brain tumor MRIs, and evaluated the performance of subsequent segmentation networks trained with the generated data. Looking at the reported results, the segmentation networks trained solely with synthetic data do not come close to those trained with real data, performance wise. Likewise, Ref. [14] proposed a combination of a variational autoencoder and a GAN, as a data augmentation framework for an image segmentation problem. Here again, the use of GANs to train downstream neural networks produced mixed (and yet more or less convincing) results.

As reported in the survey paper by [15], the application of GANs in medical imaging extends beyond image synthesis to other tasks, such as domain adaptation, classification, and reconstruction, to name a few. For these applications, the capability of GANs to generate realistic looking images, has led to a partial disregard of the usefulness of the generated medical images, or whether they hold any value compared to real data in routine clinical tasks.

In light of these publications, one might wonder how useful GANs truly are in medical imaging. In this paper, we set out to evaluate the richness and the benefit of using GAN-generated data in the context of medical imaging. We assess their performances on three datasets of different organs and different modalities.

## 2. Generative Adversarial Networks

**Figure 1:**
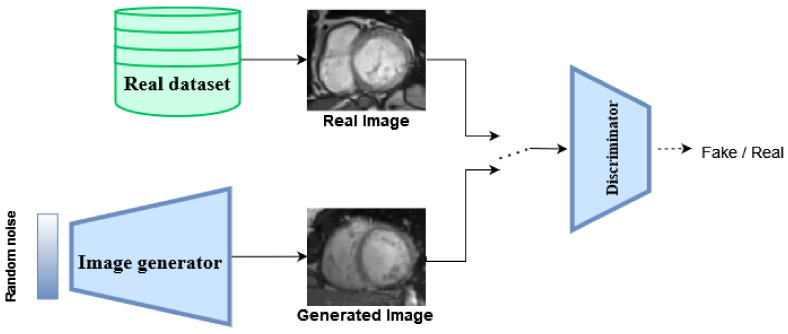
Flowchart of a traditional GAN architecture.

Adversarial networks in general, and GANs (Figure 1) more specifically, are trained to play a minimax game between a generator network, which tries to maximize a certain objective function, in tandem with a discriminator network, which tries to minimize that same objective function, hence the adversarial denomination. In their most basic formulation, GANs are trained to optimize the following loss function [1]:

(1) $$\begin{array}{c} \min_{G}\max_{D}V\left(D,G\right)=E_{x∼p\mathrm{data}(x)}\left[\log D\left(x\right)\right]\\ +E_{z∼pz(z)}\left[\log\left(1-D\left(G\left(z\right)\right)\right)\right]. \end{array}$$

here, G(z) is the *generator network*, with parameters $\theta_{G}$. It is fed with a random variable $z∼p_{z}$, sampled from a given prior distribution, that *G* tries to map to $x∼p_{\mathrm{data}}$. To achieve this, another network D (aka the *discriminator*), with parameters $\theta_{D}$, is trained to differentiate between real samples $x∼p_{\mathrm{data}}$ from a given dataset and fake samples $\hat{x}∼p_{\theta_{G}}\left(x|z\right)$ produced by the generator. In doing so, the generator is pushed to gradually produce more and more realistic samples, with the goal of making the discriminator misclassify them as real.

### 2.1. GAN Selection

The number of papers published on GANs has been growing steadily in recent years. This has been underlined by a recent survey paper [16], which reported no less than 460 references. Given this large palette of models, we based our choice on those that are the most widely adopted and/or ushered an improvement to the quality of generated images. We also selected GANs based on their ability to fit on a single 12 GB GPU, to be able to evaluate the architectures accessible to researchers with constrained computing resources.

Training GANs can be tricky. Since learning involves two opposing networks, GANs are known for suffering from several training problems, the following three being among the most widely documented.

**Convergence.** GANs (and adversarial training in general) often suffer from a lack of a defined convergence state. This is because the training process involves two networks pushing in opposite directions, without one out matching the other. This has been frequently proven to be a difficult task. For example, the generator could become too powerful and learn to fool the discriminator with faulty output. It could also happen that the discriminator reaches a 50% accuracy effectively outputting random guesses, which does not help the generator learn any meaningful information about the true data distribution.

**Vanishing Gradients.** As GANs train a generator with the output of a discriminator, whenever the discriminator significantly outperforms the generator, its loss goes to zero, pushing the retropropagated gradient to a smaller and smaller value, hence the *vanishing gradient* name. Because of that, the generator does not get enough gradient updates and sees its learning stall, to some sub-optimal solutions [17].

**Mode Collapse.** Of all the challenges that obstruct the training of powerful GANs, mode collapse might be the most difficult one to deal with. Mode collapse occurs when the generator gets stuck outputting only one (or a few) modes of the input data distribution. An example could be a generator producing images of healthy subjects, while ignoring the diseased ones. This pitfall leads to a loss of diversity in the generated datasets, that can greatly hurt the performance of subsequent networks trained with these generated data.

In regards of the aforementioned criteria and the different challenges, we selected the following GANs for our study.

#### 2.1.1. DCGAN

Deep convolutional GANs [18] were the first GANs to use convolutional layers, compared to the inital GAN which used only fully connected layers. With its simplicity, DCGAN is often the de facto baseline GAN one implements. DCGANs showed a considerable jump in image quality and training stability, while providing some useful insights on the network design (use of strided convolutions instead of pooling layers, extensive use of BatchNorm, etc.). To our knowledge, DCGAN is among the most widely implemented GANs, as of today.

#### 2.1.2. LSGAN

Least Squares GANs [19] use a different loss for the discriminator than the original GANs, which helps to alleviate certain challenges and improves the generated sample quality. LSGANs replace the cross entropy loss of the original GAN, with the mean squared error, which mitigates the vanishing gradient problem, leading to a more stable learning process.

#### 2.1.3. WGAN and WGAN-GP

Wasserstein GANs [20] were considered to be a major breakthrough, to overcome GAN training challenges. In particular, they are known to reduce the effect of mode collapse and stabilize the learning procedure. The idea is to use a Wasserstein earth-mover distance as the GAN loss function, together with some other optimization tricks, such as weight clipping and gradient penalty (WGAN-GP).

#### 2.1.4. HingeGAN (Geometric GAN)

Introduced by [21], HingeGANs substitute the original GAN loss for a margin maximization loss, which theoretically converges to a Nash equilibrium between the generator and discriminator. As for WGAN and LSGAN, HingeGAN has the sole benefit of easing the optimization process.

#### 2.1.5. SPADE GAN

Spatially adaptative denormalization (SPADE) GANs [22], are a member of the so-called *image-to-image* translation GAN family. SPADE GANs produce state-of-the-art results on a wide range of datasets, producing high quality images, perfectly aligned to a semantic input mask. SPADE GANs come as an improvement of the previously published pix2pix [23] model. SPADE GANs are considered to be the state-of-the-art conditional GANs.

#### 2.1.6. Style Based GANs

StyleGAN [24], often considered as the state-of-the-art generative neural network, introduces multiple tricks to GANs borrowed from previous works, such as progressive GANs [25], that gradually train the GAN with different resolutions, which leads to better quality and a more stable training process. StyleGAN also comes with a greatly modified generator, which includes adaptive instance normalization blocks (AdaIN), the injection of noise at every level of the network, and use an 8-layer MLP mapping function on the input latent vector $\vec{z}$.

### 2.2. Evaluation Metrics

Broadly speaking, the metrics used to quantify the effectiveness of GANs are the same as those used to evaluate traditional image synthesis tasks. This boils down to computing a similarity distance between a set of images. In their early stages, GANs were evaluated using the traditional metrics such as *Peak Signal to Noise Ratio* (PSNR) [26] or *Structural Similarity Index Measure* (SSIM) [27]. As the field advanced, more image quality metrics emerged, and became the de facto evaluation criteria, such as *Learned Perceptual Image Patch Similarity* (LPIPS) [28], *Inception Score* (IS) [29], and the *Frechet Inception Distance* (FID) [30].

The *Frechet Inception Distance* (FID), first introduced by [30], makes use of a pretrained inception network on the ImageNet [7] dataset, to assess the quality of GAN generated images. The FID is a distance between the distribution of the GAN sampled images and the real dataset used to train the GAN. Generated samples and real images are fed to the pretrained inception network and the mean and covariance of the activations in the final block, assumed to be of a Gaussian distribution, are collected for both sets, then the Frechet distance is computed between both. The FID is computed on a learned feature space and was shown to correlate well to human visual perception [28]. However, it still suffers from a number of drawbacks [31], most prominently, it suffers from a high bias [32]. In addition, FID can not detect a GAN that memorizes the training set [33].

The FID is defined as the Frechet distance between two Gaussians, as shown in Equation (2), where $N(\mu_{1},\sigma_{1})$ is the Gaussian distribution of the inception features of the real images, and $N(\mu_{2},\sigma_{2})$ the Gaussian distribution of the inception features of the generated images. In this work, we use the FID metric, as it evolves in tandem with human perception. In addition, it makes use of the original dataset to compute a distance in a learned feature space. In addition to the FID metric, we also consider the Dice score evaluation metric, obtained on a segmentation task with a U-Net network trained on the generated dataset.

(2) $$\begin{array}{c} FID\left(\left(\mu_{1},\sigma_{1}\right),\left(\mu_{2},\sigma_{2}\right)\right)={∥\mu_{1}-\mu_{2}∥}_{2}^{2}+Tr\left(\sigma_{1}+\sigma_{2}-2{\left(\sigma_{1}\sigma_{2}\right)}^{1/2}\right) \end{array}$$

## 3. Material and Methods

To make informed decisions about the usefulness of GANs in medical imaging as a source of synthetic data, we had to take into account different GANs and cover a diverse set of image modalities. In parallel, a wide range of hyperparameters had to be covered, to assess their effect on the GANs at hand.

### 3.1. Hyperparameters Search

GANs are known for their sensitivity to tweaking of the hyperparameters [33]. In order to achieve a fair comparison between the selected GANs, we covered a wide spectrum of hyperparameters (some affecting the GAN architecture), through a vast hyperparameter search, totaling roughly 500 GPU-days. We retained the best performing runs with regards to the reference metric FID, for its correlation with subjective evaluation.

Moreover, since the number of runs needed to sweep a large hyperparameter space grows exponentially with the number of hyperparameters we set to optimize over, we chose a number of sensible initial configurations for each dataset/GAN pair, mostly based on their default configuration. Table 1 lists the hyperparameters we searched over. Iterating over these hyperparameters enabled us to find the set that worked best for each GAN/dataset pair. In addition, this hyperparameter search also gave us a look at how the training stability was affected by the selected hyperparameters. Note that, some combinations were only tested for specific GANs, such as “weight clipping” for WGAN or “gradient penalty” for WGAN-GP.

### 3.2. GANs Setup

**Figure 2:**
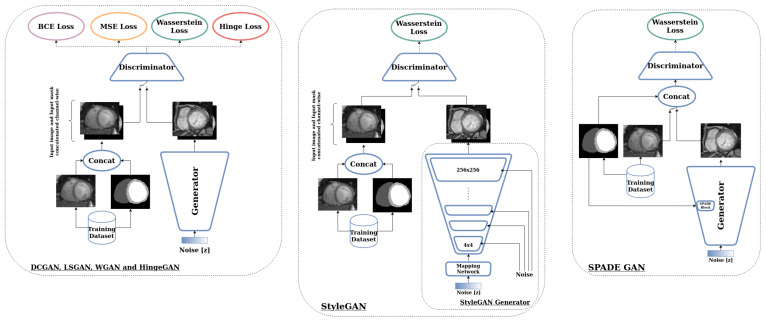
Architectures of the various GANs used.

The training of the DCGAN, LSGAN, WGAN, and HingeGAN followed the same protocol. A traditional fully convolutional network architecture, with a standard generator and discriminator composed of upconvolutions and strided convolutions, respectively, was implemented, as a basis of our DCGAN. Then the loss function was swapped, to convert it to either an LSGAN, a WGAN, or a hingeGAN. For StyleGAN and SPADE GAN, we relied on the publicly available implementations, without any change to the networks’ architecture. Figure 2 schematically summarizes the architecture of each GAN.

### 3.3. GAN Training Tricks

In order to make the GAN training process more stable, we relied on a few tricks, that have been shown to be useful in this regard.

**Label smoothing.** First applied to GANs by [29], label smoothing consists of replacing the true classification labels given to the discriminator, to a smooth value α.

**Feature matching.** Also introduced by [29], feature matching adds another objective to the generator of the GAN, which consists in minimizing a distance between the activations of the discriminator for real and generated data.

**Differentiable augmentation.** Presented by [34], differentiable augmentation imposes various types of augmentation on the fake and real samples fed into the discriminator, yielding a more stable training and better convergence.

### 3.4. GAN Evaluation in Medical Imaging

While image fidelity is fundamentally important for practitioners to deliver a good diagnostic, the visual acuity of generated images cannot be the sole marker to assess the true performance of GANs. In this paper, we want to assess how rich and diverse a synthetically generated dataset really is, in the context of medical imaging.

Thus, to verify the medical viability of GAN-generated images, we independently trained a second network, as a downstream task, on the GANs-generated datasets, and compared its results to those obtained on the original (real) datasets. In this work, we choose semantic segmentation as a downstream task to evaluate our GAN generated datasets, as it is a common task in a clinical workflow.

This assessment sets a common evaluation protocol for every GAN. This evaluation is also insightful, considering that the objective for using GANs is often to artificially increase the size of a dataset and thus provide more training data to a subsequent task [13,14]. This approach has been explored before, with GANs trained on natural images, and evaluated through a classification task [37,38].

### 3.5. Datasets

**Figure 3:**
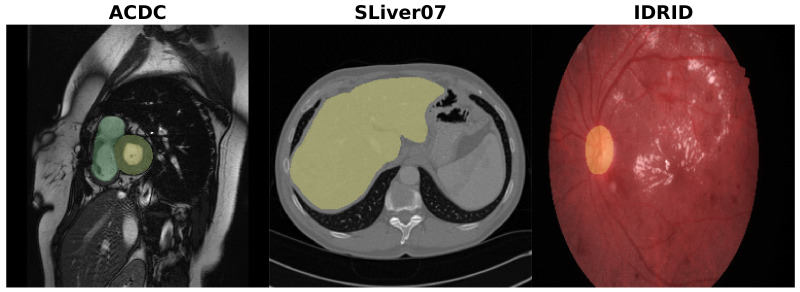
Examples of images and the segmented structures for ACDC, SLiver07, and IDRiD datasets.

To cover a good spectrum of image and medical applications, we picked three different datasets based on their imaging modalities, their organ of interest, and their size, namely, cardiac cine-MR images, liver CT, and retina imaging. These datasets offer a varied selection of data. Different dataset sizes are present, from large (SLiver07), to moderate (ACDC), to small (IDRiD). Coupled with that, different image modalities and organ shapes are considered. Figure 3 shows an example of images from each of the datasets.

#### 3.5.1. ACDC

The *Automated Cardiac Diagnosis Challenge* (ACDC) dataset [39], consists of 150 exams (100 training and 50 testing) of short-axis cardiac cine-MRI, acquired at the University Hospital of Dijon (all from different patients). The exams are divided into five evenly distributed subgroups (four pathological, plus one healthy subject groups) and further split into 100 exams (1902 2D slices) for training, with 50 exams (1078 2D slices) held out, for testing. The pixel spacing varies from 0.7 mm to 1.9 mm, with a slice spacing between 5 mm to 10 mm. The exams come with multi-structure segmentation masks for the right ventricular cavity, the left ventricular cavity, and the left ventricular myocardium, at end-diastole and end-systole times.

#### 3.5.2. SLiver07

The *Segmentation of the Liver Competition* 2007 (SLIVER07) [40] dataset, contains 40 CT volumes of the liver, enhanced with contrast agent. Most livers are pathological and include at least one tumor. The pixel spacing ranges from 0.55 mm to 0.8 mm and the inter-slice gap between 1 mm to 3 mm. The 40 CT datasets are randomly split in three groups: a group of 20 volumes for training, another group of 10 volumes for validation, and the remaining 10 volumes for testing. For our study, we only use the 20 training volumes provided with manual segmentations for the liver, which totals 4159 2D slices.

#### 3.5.3. IDRiD

The *Indian Diabetic Retinopathy Image Dataset* (IDRiD) [41], contains a total of 516 retinal fundus images of normal and pathological cases. Images are provided with disease grading ground truth for the full dataset, and segmentation masks for 81 images. We used part of the 81 images for our study, specifically, the 54 training images with the optical disc segmentation masks.

### 3.6. Dataset Generation

For our study, our selected GANs were trained on the aforementioned datasets, with the goal of synthesizing new medical data. The overarching objective of this study was to assess whether or not GANs offer a reliable framework for synthesizing realistic and diverse medical images. To examine how well GANs manage to learn the original data distribution, a large number of images was sampled from each of our trained GANs, which we later used to train a segmentation network.

**Figure 4:**
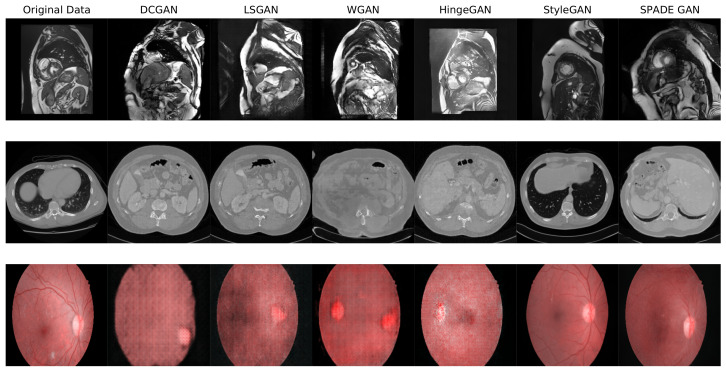
Examples of generated images for each GAN on the ACDC, SLiver07, and IDRiD datasets. The first column is an example image from the real dataset. High resolution versions of these images are available in the Supplementary Materials.

To be able to train a downstream segmentation network, the different GANs were trained on the joint distribution of the image and the mask, by concatenating the channel axis. We did so for every GAN except for SPADE, which is by nature conditioned on a segmentation mask. Once properly trained with the right set of hyperparameters, each GAN was used to generate a dataset of 10,000 images, by randomly sampling the input latent space. No further processing was performed on the generated datasets, as the objective was to gauge the quality of the *raw* images output by the GANs. Figure 4 shows some examples of images generated by each GAN on the three datasets.

## 4. Experiments and Results

This section goes through the experiments and results obtained by each GAN on each dataset.

### 4.1. Hyperparameter Search and Overall Results

**Figure 5:**
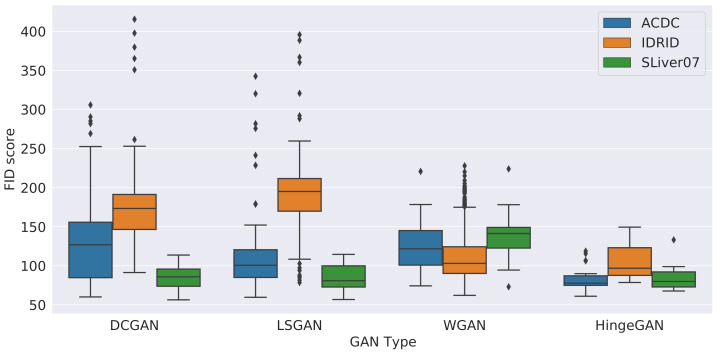
FID score for different GAN types on the IDRiD, ACDC, and SLiver07 datasets, across different hyperparameter settings.

The hyperparameter search performed on DCGAN, LSGAN, WGAN, and HingeGAN revealed interesting insights. The first one, is that some GANs are very sensitive to their hyperparameters. To underline this, the FID score obtained for every set of hyperparameters, for each GAN and each dataset, are shown in Figure 5. As can be seen, the HingeGAN has the lowest variance and, overall, the best FID score. On the other hand, DCGAN and LSGAN are overall much more sensitive to hyperparameter tweaking. This is inline with our qualitative experience, as the training of DCGAN and LSGAN often ended up producing degenerated images. SPADE and Style GAN were not included in the graph, due to the shear amount of training time they required (it took respectively 10 and 30 days to train them), but also due to their remarkable stability. Empirical evidence obtained with different hyperparameters on a few epochs, suggests that their FID variance is much lower than that of HingeGAN, hence why they ended up with top results with almost no hyperparameter tweaking.

**Figure 6:**
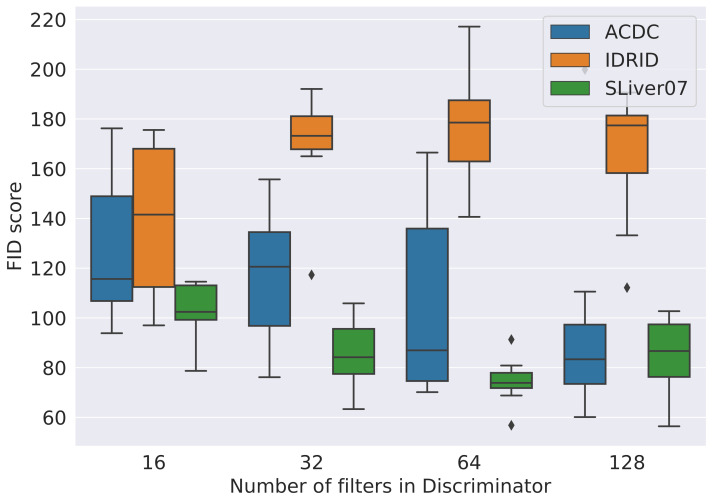
FID score for different number of filters for the discriminator of the DCGAN, LSGAN, WGAN, and HingeGAN.

Another insight comes from the impact a dataset has on the performances of GANs. As can be seen from Figure 5, the larger the reference dataset is, the better the resulting FID will be. It goes from IDRiD, the smallest datset, with FID values well above 150, to ACDC, with FIDs values roughly between 100 and 150, and finally SLiver07, the largest dataset, with most FID values being below 100. A similar trend can be seen in Figure 6, where the overall FID values for every GAN are shown against the number of convolutional filters in the discriminator network. This shows how volatile GANs can be when trained on smaller datasets, such as IDRiD. Similar plots with other hyperparameters can be found in the Supplementary Materials.

The best FID score obtained for each GAN and each dataset is shown in the third column of Table 2. Examples of generated images can also be seen in Figure 4 (and in high resolution in the Supplementary Materials). The two best models, by far, are StyleGAN and SPADE GAN. The most extreme case is for IDRiD, where a SPADE GAN got a surprising FID of 1.09 and remarkably vivid images, in Figure 4.

### 4.2. Segmentation Evaluation

The true value of the generated images was validated with a downstream segmentation network, trained on the synthetic data instead of the original (real) data. To do so, 10,000 new images were generated for each dataset and a U-Net [42] was trained to predict the segmentation mask. The architecture of the used U-Net can be found in the Supplementary Materials.

Then the U-Net was trained on the generated dataset. We predict the masks with this trained segmentation network on the test set of each of our original datasets (i.e., ACDC, SLiver07, and IDRiD). The Dice score of the prediction with the ground truth masks of the test set was computed, which will constitute our Dice score evaluation. The Dice score evaluation metric can be defined as:

(3) $$\begin{array}{c} Dice\left(Y,\hat{Y}\right)=\frac{2|Y\cap \hat{Y}|}{\left|Y|+\right|\hat{Y}|} \end{array}$$

where *Y* is the ground truth segmentation mask and $\hat{Y}$ is the predicted segmentation mask.

The last column of Table 2 contains the Dice score obtained on the *real* test set of each dataset. Unsurprisingly, as suggested by the FID scores, StyleGAN and SPADE GAN achieve the highest Dice scores on all the datasets, with StyleGAN reaching 87% Dice on the ACDC dataset, 2% less than when training with the original data.

These results reveal three important things about GANs in medical imaging. First, simpler models such as DCGAN, LSGAN, WGAN, and HingeGAN perform systematically poorly on every dataset, despite an intensive hyperparameter search. This suggest that these models might be ill-suited for medical imaging applications.

Second, despite their visual similarity, GAN-generated datasets do not have the same richness as real datasets. This is illustrated by the fact that, despite being trained on far more images, none of the GAN Dice scores equal or outperform the ones obtained on the original datasets. Moreover, the generated datasets, when used as augmentation data, achieve similar performance to traditional augmentation techniques (rotations, shifts, flips), illustrated by the Dice score of training with a mix of the original data and generated data, and the augmented original data only.

Third, while the FID score is a good proxy to distinguish the *best* methods from the *least effective* ones, it does not correlate well with an application score, such as the Dice score. For example, the FID score of 29.06 of StyleGAN on SLiver07 suggests that the produced images are much more accurate than those of SPADE GAN (FID = 47.62). However, the resulting Dice scores show that SPADE GAN is significantly better than any other model. A similar comment can be made for IDRiD and ACDC, as StyleGAN and SPADE GAN got similar Dice scores but very different FIDs. As for the FID score of 1.09 obtained by SPADE GAN, the associated 82% Dice score suggests that the network has most likely memorized the training set. This might be attributed to the small size of the IDRiD dataset, as well as to the simple shape of the input segmentation mask.

**Figure 7:**
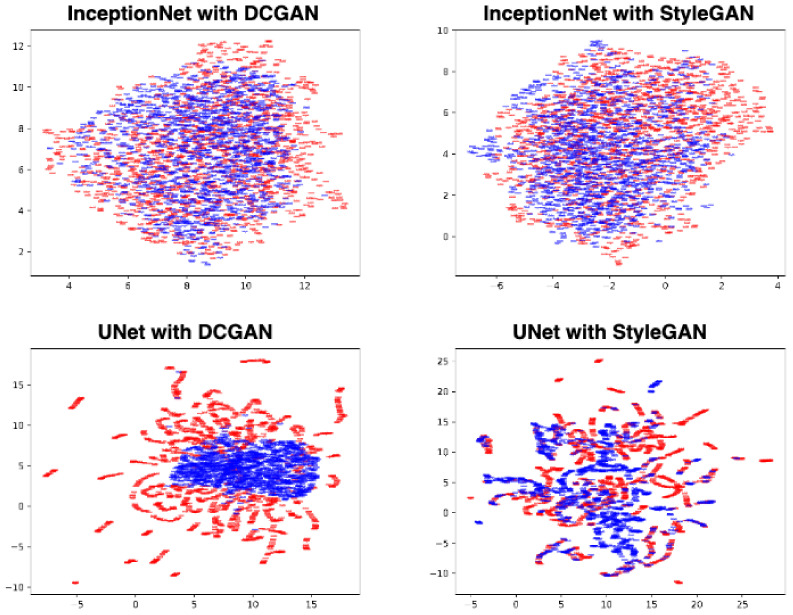
Comparison of UMap projection of activations of images generated by a DCGAN, and others generated by a StyleGAN, with an InceptionNet trained on ImageNet (**top row**), and a U-Net trained on the original dataset (**bottom row**). Red points: real images; blue points: generated images.

To further analyze whether the FID score is a reliable medical imaging metric, we plotted the InceptionNet latent space of the generated images obtained with the most and the least effective GANs, i.e., DCGAN and StyleGAN (c.f. top row of Figure 7, plots were obtained with UMap [43]). In parallel, we plotted the U-Net latent space for the same images and the same GANs (cf. bottom row of Figure 7). While the red and the blue InceptionNet scatter plot distributions are very similar for DCGAN and StyleGAN, the U-Net ones reveal much more distinctive patterns. Indeed, the U-Net distributions of StyleGAN follow very similar distributions (hence suggesting that the synthetic images of StyleGAN are visually very close to those of the original dataset), while the ones from DCGAN show a clear case of mode collapse. This underlines a fundamental limit of the FID metric: since the InceptionNet was trained on ImageNet (a non-medical dataset), its use in medical imaging must be made with great care.

### 4.3. Visual Turing Test

Considering how realistic looking some of the GAN-generated images are, we asked four medical experts, each with more than 15 years of experience in cardiology, to classify fake and real cine MRI images generated by StyleGAN and from the ACDC dataset. Each expert was shown 100 images, consisting of a 50/50 mixture of real and synthetic images, and was asked to classify it based only on their visual appreciation. The accuracy of the classification performed by the experts was equal to 60% (+/−10%). This result shows how visually accurate the generated images are.

## 5. Discussion

In this section, we go through the aspects that play a major role in the process of training GANs with medical data.

### 5.1. Training Volatility

Throughout this work, the training instability of GANs was a recurrent theme, underlying how slight hyperparameter adjustments can considerably affect the training process. In contrast, GANs were not equally sensitive to the selected hyperparameters. While it is true that DCGAN and LSGAN showed the highest variability, it came to be easier to train WGAN and HingeGAN, which were less sensitive to hyperparameter selection.

Moreover, even though the state-of-the-art GANs, such as SPADE and StyleGAN, seem to be the only viable pick to produce images of high quality, they still suffer from long training times and can sometimes lead to overfitting and “Memory GAN”, i.e., a GAN that outputs the training set.

Likewise, in the case of the smaller GANs, finding the right set of hyperparameters was not always simple. To illustrate this point, we went through a total of 1500 training runs with different hyperparameter combinations. Most of the runs led to models that could not generate meaningful images, while the remaining runs did not always fair well when evaluated with the FID, or through the image segmentation task. Concurrently, although a considerable amount of hyperparameters were explored, we did not have enough GPUs to go through a GAN architecture search, which could have provided better performance.

### 5.2. FID and Image Quality

We relied on the FID score to monitor the training of the GANs. We also compared FID to a domain specific evaluation (segmentation Dice score). This process, enabled us to better understand to what extent an FID metric, optimized for natural images, can be used in medical imaging. Our results reveal that the FID score continuously improves as the training of any GAN moves forward. In contrast, the FID score could not be consistently relied on as a measure of the image quality when used as training input for subsequent tasks. Table 2 clearly shows that a lower FID score, does not always yield better performance on a subsequent task of image segmentation. These results make it interesting to ask whether metrics grounded in domain specific knowledge, could help make GANs easier to evaluate and compare.

### 5.3. Data Scale

When comparing the results on the three datasets, an important trend related to the performance of the GANs and the data is visible. When the size of the input dataset is exceedingly small, as is the case for the IDRiD dataset in our study, the expected benefit of training a GAN to increase the dataset size, quickly dissipates, as they often overfit, which can have an adverse effect on the subsequent task. In parallel, when the input dataset is highly unbalanced, portrayed by the SLiver07 dataset in our study, with only 5% foreground pixels, the trained GANs can further exacerbate this imbalance, as they will ultimately learn the underlying biases of the training data. The variety in the input dataset matters as much as the number of data points. Depending on the variety of the data, the GAN can overfit to a single mode in the input distribution or truly learn the overall distribution. Moreover, most of the prevalent GAN architectures deal with 2D images, which is not necessarily the best format to train with when dealing with medical imaging data, as it might have been acquired in 3D. This might further explain the poor performance shown on the liver CT dataset.

### 5.4. Compute Scale

It should be kept in mind that training a GAN is often computationally intensive (typically because it involves two or more networks), and requires a large amount of memory. In addition, training GANs requires a lot of hyperparameter tuning, which may or may not lead to better results when considering the downstream tasks the generated data is intended for. This also affects more sophisticated GANs which, despite their good performances, which can fool medical experts, require large computing resources to train. For example, the StyleGAN took roughly 30 days to train on the ACDC dataset, with an NVidia Titan V GPU, with 12 GB of memory. Yet, StyleGAN did not always offer a guarantee to the usefulness of the generated samples (Dice score of 0.36 for StyleGAN on the SLiver07 dataset).

### 5.5. Medical Worth

As there is no automated objective way to assess whether a medical image conveys the information for the diagnosis it is intended for, we based our analysis on a proxy task, that aims to mimic the process for which a dataset is created, and compared its performance to that of the original data. Here the proxy task is the evaluation of the segmentation performed by a U-Net, and the results are evaluated by the Dice score. The results show that, although most of the images generated by the tested GANs fail in reaching the baseline performance, some of the more advanced ones manage to close the gap. However, when subjectively assessing the images generated by the larger GANs, we can still see that they exhibit a remarkable degree of complexity and quality. This might be related to the smaller scale of the datasets in medical imaging, and the difference in their nature with the original datasets for which most of the GANs were tailored. Likewise, a considerable amount of the medical data is acquired in a 3D fashion and voxel wise, e.g., CT. Typical GANs might not capture the full extent of the medical information, when trained solely on 2D views. Indeed, this makes exploring GANs specially made for medical data an interesting research avenue, and could lead to an improvement in quality and ultimately clinical usability.

## 6. Conclusions

Currently the use of deep learning approaches in medical image analysis remains hindered by the limited access to large annotated datasets. To address this limitation, we have probed both the limitations and promising aspects of generative adversarial networks as medical image synthesis tools, through an experimental approach on three different datasets. As a result, GANs’ effectiveness as a source of medical imaging data was found to not always be reliable, even if the produced images are nearly indistinguishable from real data. Tangentially, the results point to the fact that traditional metrics used to evaluate GANs are less robust than task based evaluations.

All in all, this study should drive more research on GANs that take into account the different subtleties of medical data and hopefully lead to better generative models.

## Figures and Tables

**Table 1:** List of the different hyperparameters optimized over.

Hyperparameters	Values
Differentiable augmentation [34]	TRUE/FALSE
Activation fn of discriminator	ReLU/LeakyRelu/Elu/Selu
Activation fn of generator	ReLU/LeakyRelu/Elu/Selu
Normalization layer of discriminator	BatchNorm [35]/InstanceNorm [36]
Normalization layer of generator	BatchNorm [35]/InstanceNorm [36]
Number of filters of discriminator	16/32/64/128
Number of filters of generator	16/32/64/128
Use spectral norm for discriminator	TRUE/FALSE
Use spectral norm for generator	TRUE/FALSE
Weight initialization function	Normal/Xavier/Xavier Uniform/Kaiming He
Weight initialization gain	0.01/0.02/0.1/1.0
Gradient penalty loss weight (WGAN-GP only)	0/0.1/1.0/10.0
Weight clipping value (WGAN only)	0/0.01/0.1
Feature matching loss weight	0/1.0/10.0
VGG loss weight	0/1.0 /10.0
Learning rate	0.00004/0.00005/0.0001/0.0002/0.001
Use of label smoothing [29]	TRUE/FALSE
Use of data augmentation	TRUE/FALSE

**Table 2:** FID and U-Net Dice score for different GANs on the ACDC, IDRiD, and SLiver07 datasets. Best score per metric for each dataset are highlighted in bold.

Dataset	GAN	FID Score	U-Net Dice Score
	Original Data	–	**0.89**
	Augmented Original Data	–	**0.90**
	DCGAN	60.12	0.30
	LSGAN	59.65	0.39
ACDC	WGAN	74.30	0.70
	Hinge GAN	61.00	0.63
	SPADE GAN	41.54	0.86
	StyleGAN	**24.74**	**0.87**
	Orig. Data + SPADE GAN	–	**0.90**
	Orig. Data + StyleGAN	–	**0.90**
	Original Data	–	**0.83**
	Augmented Original Data	–	**0.84**
	DCGAN	91.34	0.29
	LSGAN	78.61	0.20
IDRiD	WGAN	62.12	0.72
	Hinge GAN	78.61	0.69
	SPADE GAN	**1.09**	**0.82**
	StyleGAN	23.72	0.80
	Orig. Data + SPADE GAN	–	**0.84**
	Orig. Data + StyleGAN	–	**0.84**
	Original Data	–	**0.72**
	Augmented Original Data	–	**0.70**
	DCGAN	56.41	0.14
	LSGAN	56.82	0.15
SLiver07	WGAN	73.11	0.16
	Hinge GAN	67.69	0.15
	SPADE GAN	47.62	**0.61**
	StyleGAN	**29.06**	0.36
	Orig. Data + SPADE GAN	–	**0.71**
	Orig. Data + StyleGAN	–	**0.71**

## Data Availability

All the data used in this study is part of already published public datasets: ACDC [39], SLiver07 [40] and IDRID [41].

## Supplementary Materials

The following supporting information can be downloaded at: https://www.mdpi.com/article/10.3390/jimaging9030069/s1, Figure S1: FID for different discriminator normalization layers, Figure S2: FID for different generator normalization layers, Figure S3: FID for whether discriminator uses spectral normalization or not. Figure S4: FID for whether we use differentiable augmentation or not., Figure S5: Examples of generated samples, Figure S6: More Examples of generated samples, Figure S7: Architecture of the U-Net used for the segmentation task.
