# Supplementary material for: GANs for Medical Image Synthesis: An Empirical Study
Source: J Imaging. 2023 Mar 16;9(3):69. doi: 10.3390/jimaging9030069 (PMC10055771; doi:10.3390/jimaging9030069)
Supplement: Supplementary file 1 [file jimaging-09-00069-s001.zip › jimaging-2150191-supplementary.pdf]

Article

GANs for Medical Image Synthesis: An Empirical Study

Youssef Skandarani [1,2,*], Pierre-Marc Jodoin [3] and Alain Lalande [1,4]

[1] University of Bourgogne Franche-Comte, 21000 Dijon, France
[2] CASIS Inc., 21800 Quetigny, France
[3] Department of Computer Science, University of Sherbrooke, Sherbrooke, QC J1K 2R1, Canada
[4] Department of Medical Imaging, University Hospital of Dijon, 21079 Dijon, France
* Correspondence: youssef.skandarani@outlook.com;

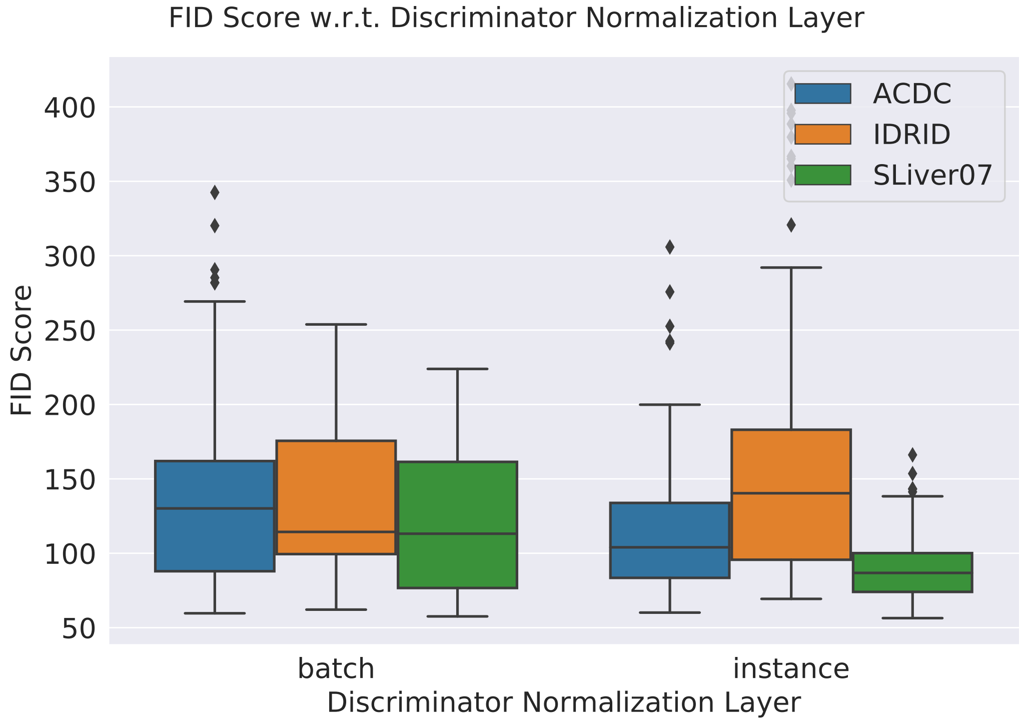

Figure S1. FID for different discriminator normalization layers

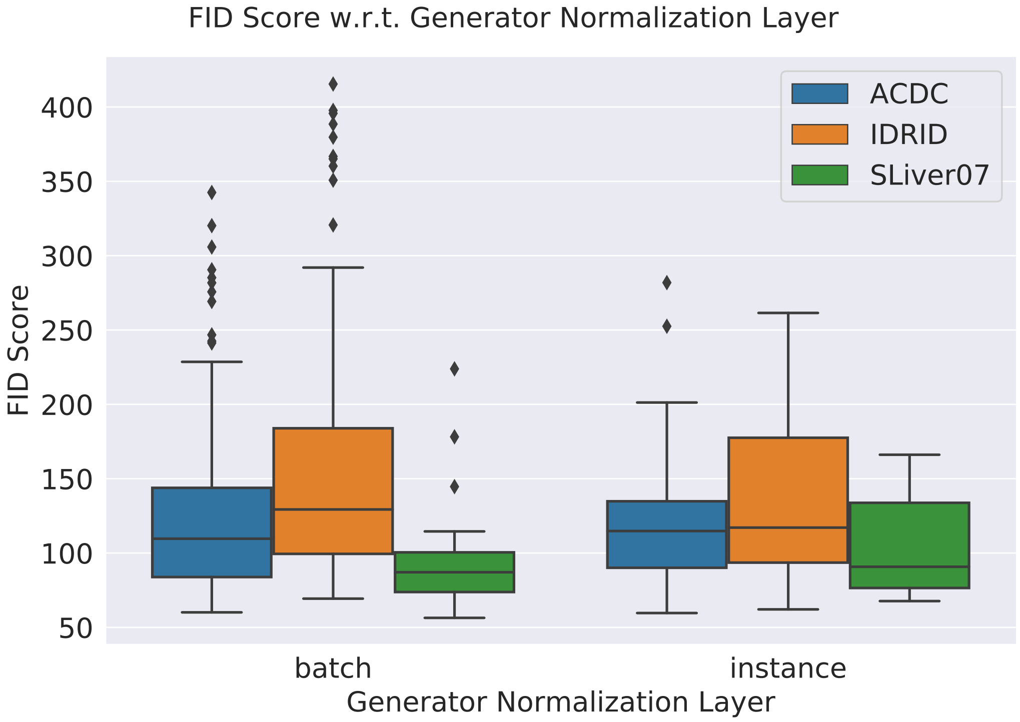

Figure S2. FID for different generator normalization layers

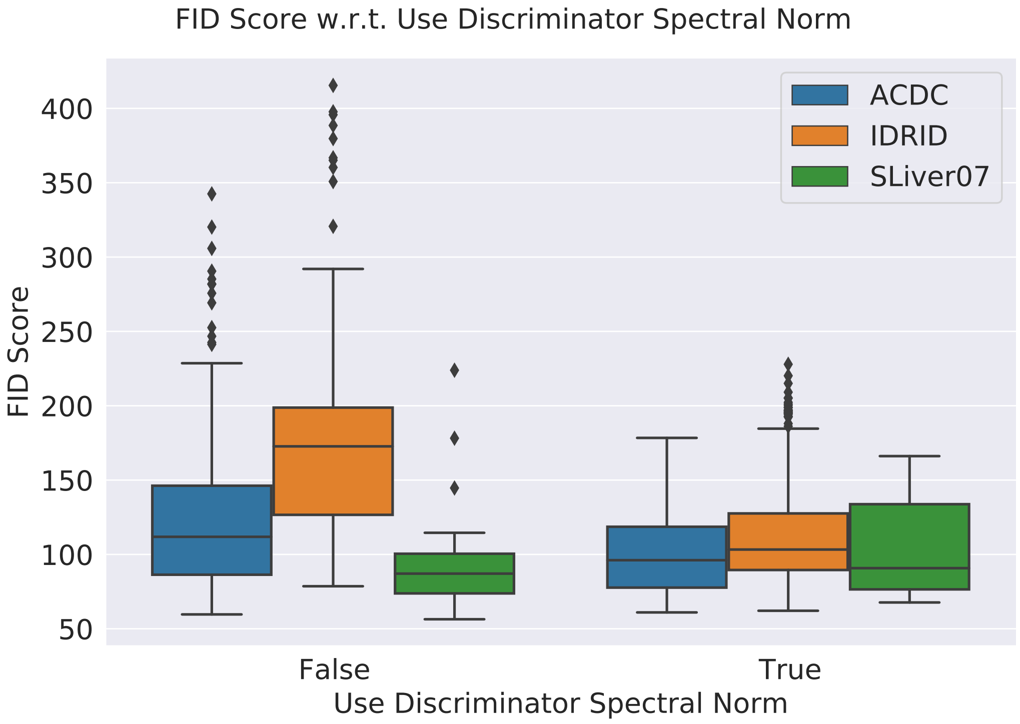

Figure S3. FID for whether discriminator uses spectral normalization or not.

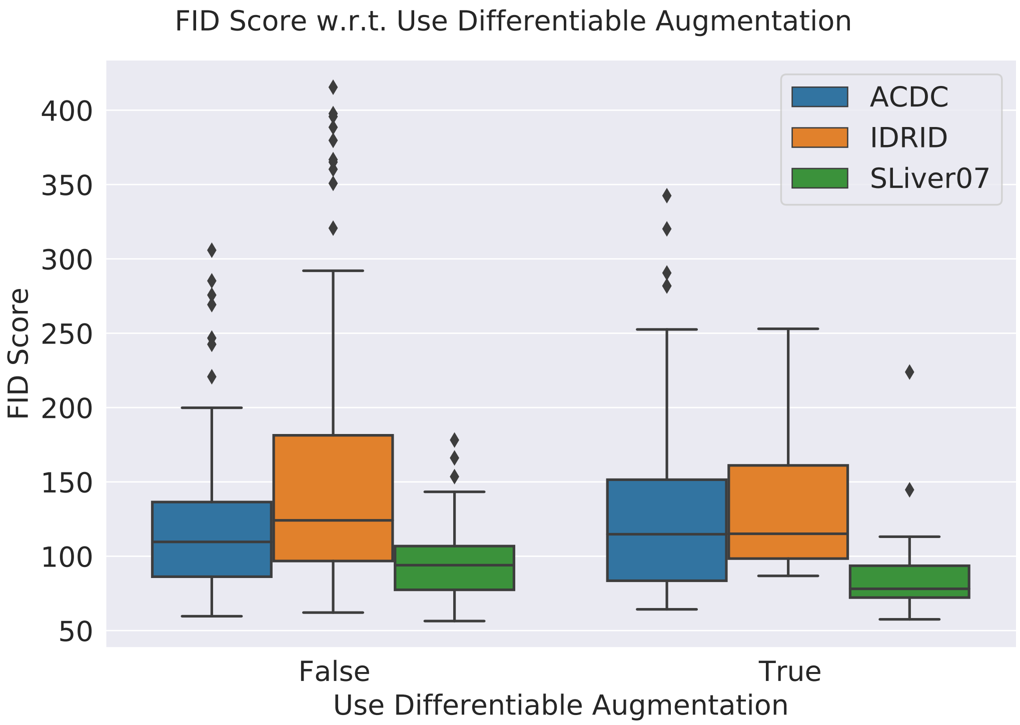

Figure S4. FID for whether we use differentiable augmentation or not.

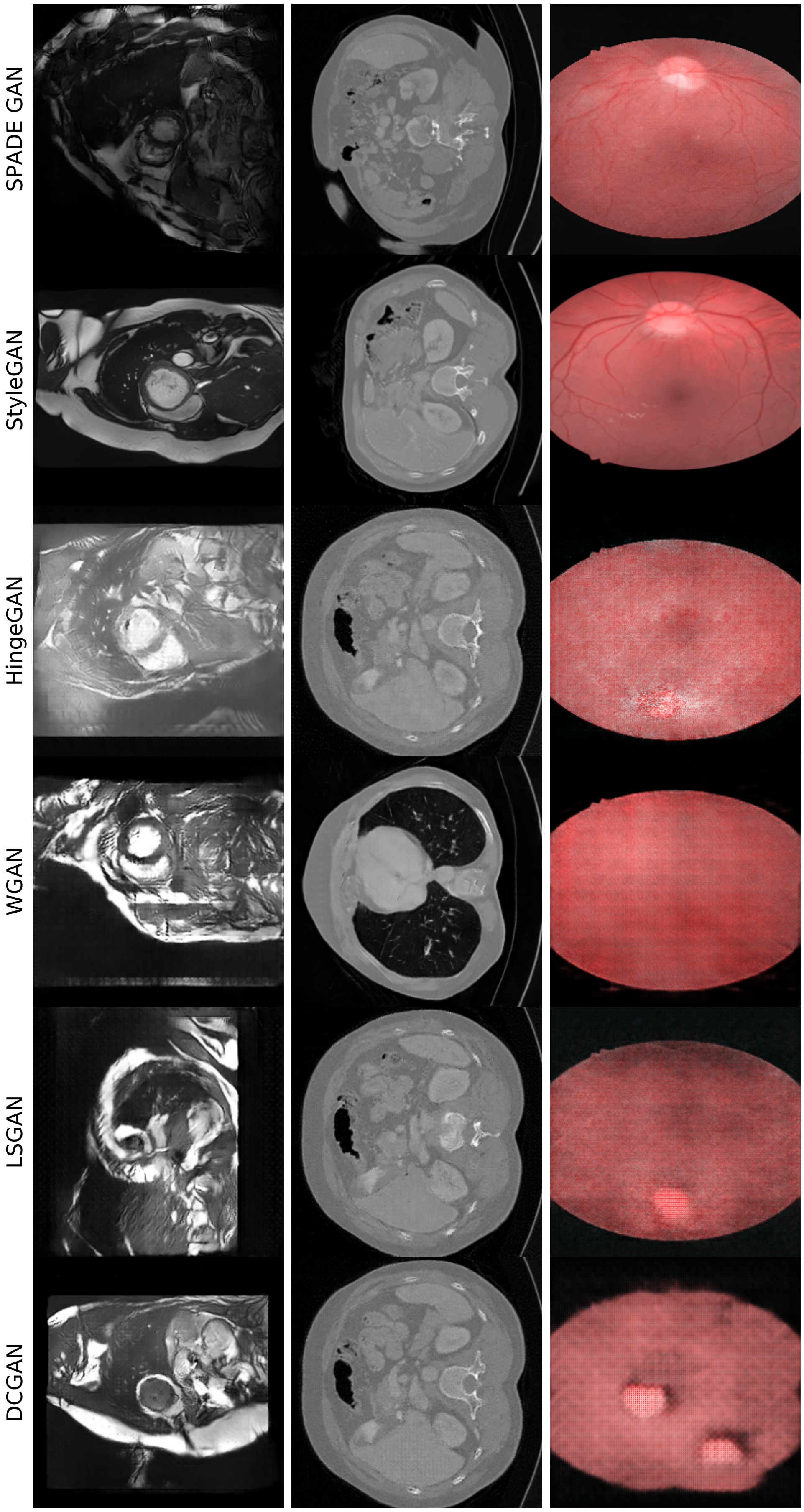

Figure S5. Examples of generated samples

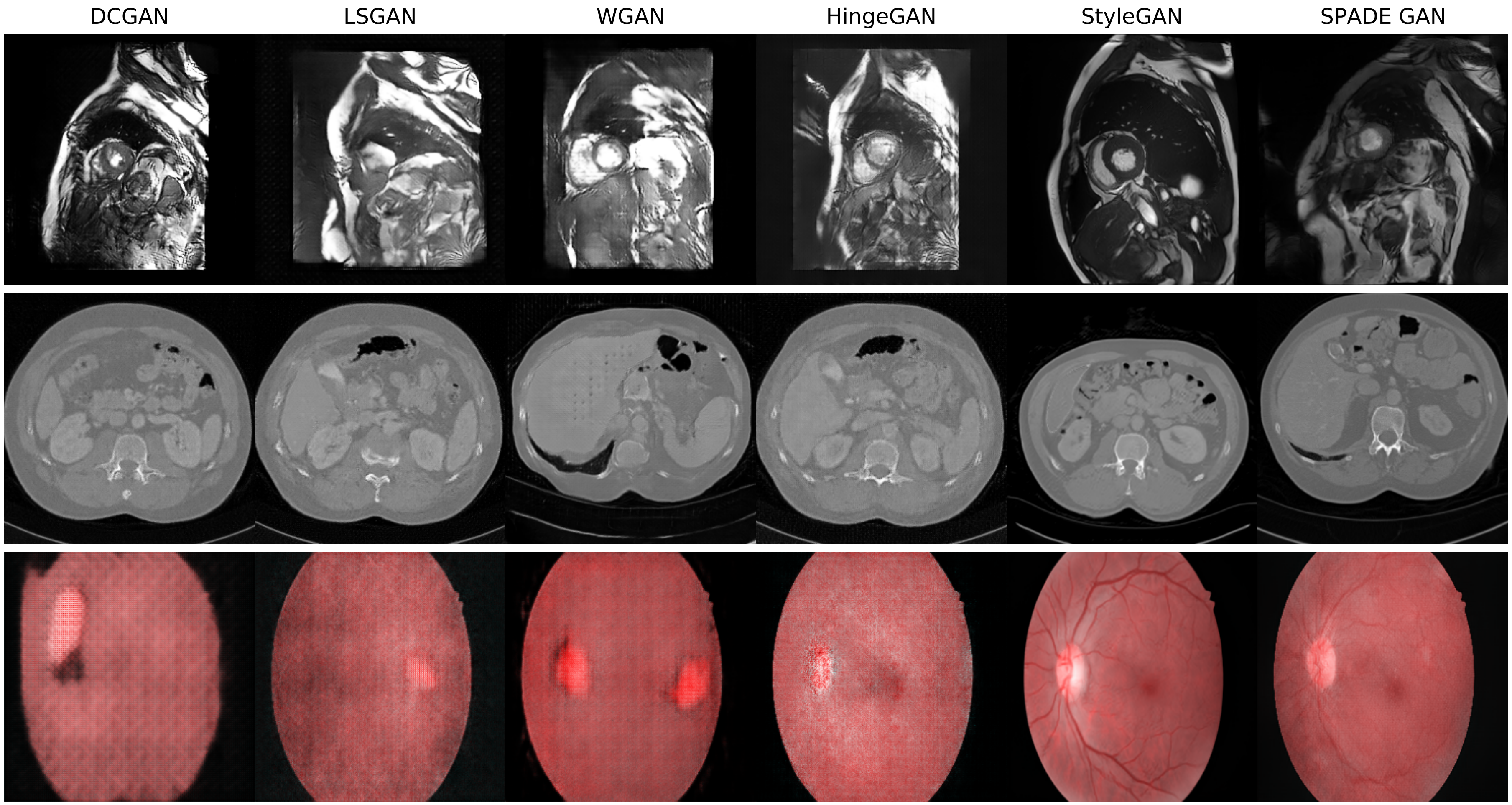

Figure S6. More Examples of generated samples

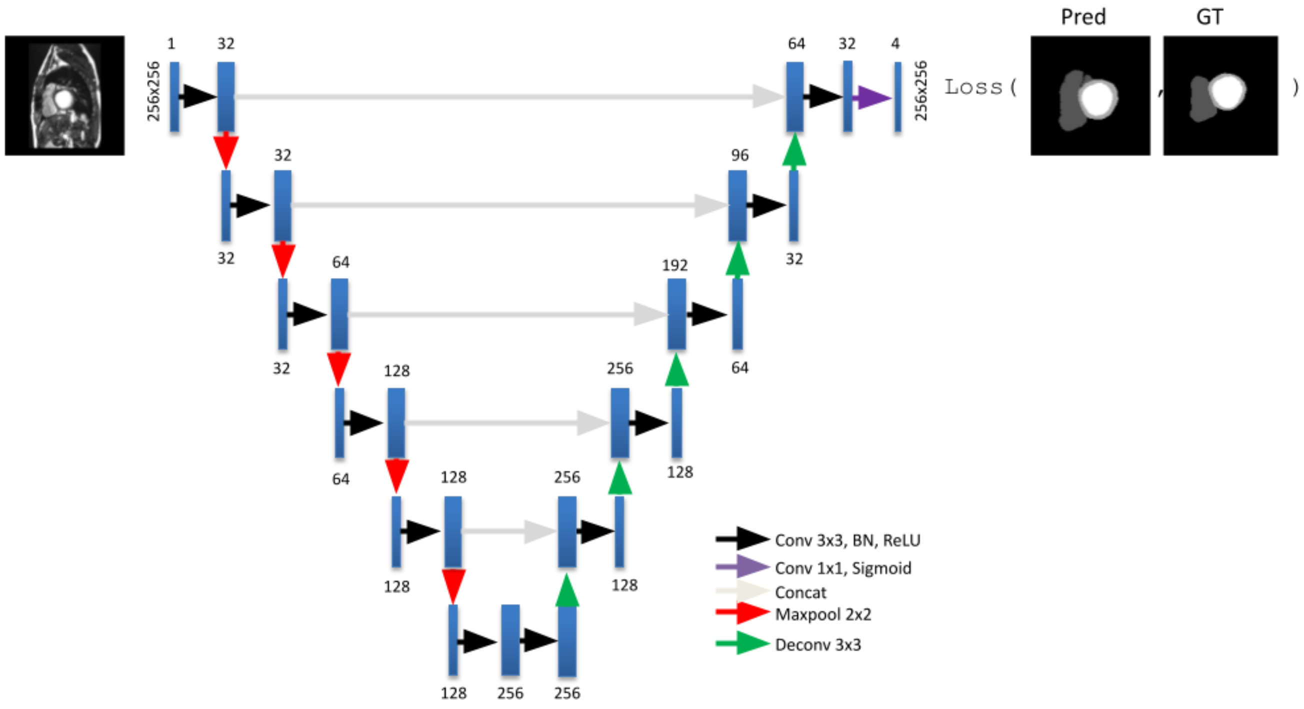

Figure S7. Architecture of the U-Net used for the segmentation task.
